# Stimulatory effects of smoke solution and biogas digestate slurry application on photosynthesis, growth, and methylation profiling of *solanum tuberosum*

**DOI:** 10.1080/15592324.2024.2336724

**Published:** 2024-04-10

**Authors:** Rafi Ullah Khan, Irfan Ullah, Ghazal Khurshid, Sultan Suboktagin, Abdul Rehman Khan, Iftikhar Zeb, Zahid Ahmad Khan, Muhammad Jamil, Eui Shik Rha, Hayssam Muhammad Ali, Raza Ahmad

**Affiliations:** a Department of Biotechnology, COMSATS University Islamabad, Abbottabad, Pakistan; b Department of Biotechnology and Genetic Engineering, Kohat University, Kohat, Pakistan; c Department of Wellbeing Resources, Sunchon National University, Sunchon, South Korea; d Department of Botany and Microbiology, College of Science, King Saud University, Riyadh, Saudi Arabia

**Keywords:** Biostimulants, biogas digestate slurry, methylation, plant-derived smoke, photosynthesis, pigments

## Abstract

Biostimulants are obtained from various sources like plants, animals, microorganisms, and industrial by-products as well as waste material. Their utilization in agriculture practices is being increased that is giving positive results. The purpose of the current study was to use plant-derived smoke (SMK) solution and biogas digestate (BGD) slurry as biostimulant to elucidate their impact on potato (*Solanum tuberosum*) performance. The experiment was conducted in lab as well as field conditions, and SMK and BGD solutions were prepared in varying concentrations such as SMK 1:500, SMK 1:250, BGD 50:50, and BGD 75:25. Foliar applications were performed thrice during experiments and data were collected related to photosynthesis, growth, pigments, and genome-wide methylation profiling. Net photosynthesis rate (*A*) and water use efficiency (WUE) were found higher in SMK- and BGD-treated lab and field grown plants. Among pigments, BGD-treated plants depicted higher levels of Chl a and Chl b while SMK-treated plants showed higher carotenoid levels. Alongside, enhancement in growth-related parameters like leaf number and dry weight was also observed in both lab- and field-treated plants. Furthermore, DNA methylation profile of SMK- and BGD-treated plants depicted variation compared to control. DNA methylation events increased in all the treatments compared to control except for SMK 1:500. These results indicate that smoke and slurry both act as efficient biostimulants which result in better performance of plants. Biostimulants also affected the genome-wide DNA methylation profile that resultantly might have changed the plant gene expression profiling and played its role in plant responsiveness to these biostimulants. However, there is need to elucidate a possible synergistic effect of SMK and BGD on plant growth along with gene expression profiling.

## Introduction

In recent era, the global climatic changes and rising population have elevated the challenges for agriculture sector to improve the plant productivity that help feed the masses.^1^ The challenges in improving plant productivity can be due to conventional agronomic practices and expensive agrochemicals.^2^ The agrochemicals or synthetic fertilizers have been employed in the agriculture over the years to increase the growth and yield of plants.^3^ There is a need to adopt novel agricultural practices that not only enhance utility of these agrochemicals but are also resource-friendly.^3^ One of the resource efficient approaches is to employ the bio-based agents (biostimulants) that can positively stimulate the physiological processes of plants.^4^

Biostimulants provide potential novel and an eco-friendly approach that can help improve the plant growth and productivity.^5^ They enhance the nutrient uptake, crop quality and also help them survive in stress conditions such as salinity, varying temperature, and water scarcity.^4,6,7^ Biostimulants are classified on the basis of mode of application (soil or foliar), natural sources and the method through which they were produced.^8^ They are derived from wide array of biological substances such as microorganisms, algae, plants, animal, seaweed, humic substances, and other industrial by-products.^1,9^

Utilization of waste or inexpensive materials as source of biostimulants paves way toward recycling and reuse, thereby improving circular bioeconomy.^10^ By-products produced by utilizing the waste materials include vermicompost, composted urban waste, sewage sludge, protein hydrolyzate, and chitin/chitosan derivatives, to name a few.^7^ Slurry obtained after anaerobic digestion of farmyard manure in biogas production is also considered as beneficial organic fertilizer.^11,12^ It is cost-effective as farmers can obtain the slurry by employing agriculture waste and avoid the impact of synthetic fertilizers such as soil or water pollution.^12^ Biogas slurry or digested biogas effluent contains macro- and micro-nutrients especially potassium (K: 1%), phosphorous (P: 1.1%), and nitrogen (N: 1.5%) that help improve the soil health and crop yield.^12^ The biogas slurry application has shown improvement in soil rhizosphere nutrient mixture with higher levels of N, P, and K in *Camellia oleifera* fruit content.^13^ Currently, biogas slurry application is being reported in different crops including wheat, rice, maize, lettuce, cabbage, and apple wherein significant improvement in biomass and quality has been observed.^14^

Plant-derived smoke solution has been credited as a promising biostimulant that improved plant pre- and post-germination parameters effectively.^15^ Smoke has been identified as stimulating agent of germination in fire-exposed environments.^15^ Smoke contains mixture of compounds but the most active are the karrikins (KAR) and cyanohydrin that help in early germination and also promote plant growth and biochemical mechanisms.^16^ Reportedly, plant-derived smoke has enhanced the photosynthetic rate, total nitrogen content, total soluble sugars, and proteins in treated plants.^17^ In addition to normal growth parameters, smoke water also helps reduce the stress effects in plants.^18^ *Moringa oleifera*-derived smoke water reduced the impact of cadmium stress and improved the physiological and biochemical parameters of rice.^19^ In another study, smoke water obtained from rice straw has modulated the oxidative stress and subsequently enhanced root growth.^20^ Furthermore, enhancement in expression of stress responsive genes and enzymatic activity has also been observed in wheat under salt stress.^21^

Keeping in view the importance and utility of these biostimulants in improving plant traits, they can be a vital component of integrated plant growth strategy. This study elucidates the effects of foliar application of smoke and slurry solutions on the growth of potato plants. The application of these by-products significantly improved photosynthesis, growth, water use efficiency, and pigment contents of potato plant. The smoke and slurry application has also altered the genome-wide methylation pattern in treated plants.

## Materials and methods

### Plant material, growth conditions and treatments

*Solanum tuberosum* (Cv. Sarpo *mira*) tubers were obtained from Hazara Agriculture Research Center (Abbottabad, KP, Pakistan). For lab experiment, the tubers were planted in pots and after germination, the stem cuttings were sterilized and cultured into MS media^22^ supplemented with 3% sucrose (phytotechnology, USA). After getting the contamination-free plants, the apical stem sections of these plants were further sub-cultured to obtain uniform size plants. After two weeks of subculturing, the rooted plants were shifted to pots containing peat moss and vermiculite (2:1). The plants were acclimatized for a week and then allowed to grow in a growth room with temperature 25 ± 2°C and 16/8 h (light/dark). After 4 weeks of growth, the plants were treated with smoke (SMK) solution and biogas digestate (BGD) slurry via foliar application. The application was repeated twice after intervals of 10 days. For field experiment, tubers of similar size were selected and planted in rows in the experimental field of COMSATS University Islamabad, Abbottabad campus (KP, Pakistan). The experiment was conducted in randomized complete block design such that five tubers were planted in each replicate. The irrigation was performed in intervals as required and no synthetic fertilizer was utilized in the experiment. After 5 weeks of germination, the plants were treated with SMK and BGD through foliar application, and the treatment was repeated twice after intervals of 10 days.

The smoke solution was prepared from *Cymbopogon jwarancusa* as reported.^23^ The concentrated smoke solution was diluted into the ratio (v/v) of 1:500 and 1:250. The BGD slurry was obtained from bioproduct lab, COMSATS University Islamabad, Abbottabad campus. The slurry was filtered by a filter paper to remove the debris and diluted in concentrations (v/v) of 50:50 and 75:25. The dilutions were made with distilled water. 10 ml of each solution was applied through handheld spray machine on plants grown in lab while for field grown plants 15 ml was applied. The control plants were sprayed with distill water in both experiments (lab and field). While spraying the plants, it was made sure that spray drift should not reach the non-target plants.

The characterization of slurry was also performed measuring total solids (TS), volatile solids (VS), chemical oxygen demand (COD), and pH through standard APHA methods (Table 1).^24^ Total reducing sugars were measured according to the method reported previously.^25^ The nutrient content of the digestate samples was determined according to the reported methods.^26^ The nitrogen content was measured as NH_4_^+^ while phosphorous content was determined as P_2_O_5_.^27^

**Table 1.** Physicochemical characterization of digestate.

Parameters	Unit	Digestate
Total solids	%	4.16 ± 0.008
Volatile solids	%	3.96 ± 0.010
N measured as NH_4_^+^	mg/L	242.9986 ± 1.85
P measured as P_2_O_5_	mg/L	95.626 ± 6.20
Chemical oxygen demand	mg/L	10.836 ± 2.59
pH	-	7.26 ± 0.42
Total Reducing Sugars	mg/L	742 ± 224.33

### Data collection from lab and field grown plants

#### Measurement of photosynthetic parameters

After completion of treatments and an interval of 10 days (65 days of growth), the photosynthetic parameters including net photosynthesis rate (*A*), intercellular CO_2_ (C_i_), transpiration rate (E), and stomatal conductance (g_s_) were measured using portable gas exchange system IFL (ADC Bioscientific Ltd., Hoddesdon, UK). The system parameters were kept similar to literature reported.^28^ The water use efficiency was also calculated according to literature.^29^

#### Measurement of chlorophyll and carotenoid content

For quantification of chlorophyll a (Chl a) and chlorophyll b (Chl b) and total carotenoids, 1 g fresh weight of leaf was taken, mixed with 5 ml of 80% acetone and ground well with the help of pestle and mortar. It was then centrifuged at 10,000 rpm for 10 min. The supernatant (0.5 ml) was mixed with chilled acetone (4.5 ml). The absorbances were recorded at λ = 663 nm, λ = 645, and λ = 470 nm for chlorophyll a, chlorophyll b, and total carotenoids, respectively, using T80+UV/Vis Spectrophotometer (PG Instruments Ltd., UK). The quantification for these parameters was made as reported in literature.^30^

#### Evaluation of growth-related parameters in lab and field plants

Growth-related parameters were measured in lab- and field-grown potato plants which include number of leaves per plant, leaf area, dry weight, and tuber weight. The experiment was set in three replicates. Leaf area was measured in 65 days old plants by taking third fully expanded leaf from apex using graph paper. Manually leaf was drawn on graph paper containing 1 cm^2^ squares, fully covered squares were considered as 1 cm^2^ of area while half-covered squares were considered as half. The number of leaves per plant were counted in the lab experiment after 90 days; however, in field experiment, the leaf number was recorded at maturity (115 days). Potato has compound leaves so each leaf (including leaflets) was considered one. In the lab experiment, after 90 days of growth, the plants were uprooted and dried in drying oven at 72°C for 3 days. At maturity level, the field grown plants were harvested and dried in similar manner. Then, the plant dry weight was measured using weighing balance (Pioneer OHAUS, USA). In the field grown plants, tubers were collected after harvesting, washed to remove the soil, and weighed.

#### Analysis of soluble sugars in treated plants

For soluble sugars, leaves were collected after 8 h of light in lab and midday in field. Briefly, the leaves were crushed and then extraction was done by mixing them (50 mg) in hot ethanol. The mixture was then incubated in water bath for 10 min at 95°C. The extraction was done three times and then the supernatant was further used for soluble sugars. The absorbance was taken at 490 nm using T80+UV/Vis Spectrophotometer (PG Instruments Ltd., UK).^29,31^

#### Genomic DNA extraction and methylation profiling

After evaluating physiological, biochemical, and morphological parameters, methyl-sensitive amplification polymorphism (MSAP) analysis was also performed on field grown treated and control plants to determine the impact of treatments on DNA methylation profile.^32^ First, genomic DNA was extracted from control and treated plants using DNA extraction protocol.^33^ Briefly, genomic DNA (100 ng) was digested with the *Eco*RI for 2 h at 37°C. Then, *Eco*RI was inactivated by incubating the samples at 65°C for 20 min. Afterward, the digested DNA was equally aliquoted into two separate eppendorf tubes and was independently digested with *Msp*I and *Hpa*II for overnight at 37°C. The restricted samples were then ligated with *Eco*RI/ *Hpa*II linkers using T4 DNA ligase (kept at 4°C overnight).

The aliquot was then used for PCR analysis which was carried out through PTC-100 thermal cycler (Bio-Rad, California, USA) using three primer pairs as reported.^32^ According to reported study, the PCR conditions, PCR product visualization, and data scoring were performed.^32^

### Statistical analysis

The data related to physiological, morphological, and biochemical parameters were analyzed by one-way ANOVA, and grouping was performed using LSD method at *p* < 0.05 level. Principal component analysis (PCA) was performed to analyze the data grouping and variance patterns. All statistical analyses were performed using Minitab software version 17.

## Results

### *Exogenous treatments improved the photosynthetic rate (*A*) in lab and field conditions*

**Figure 1.**
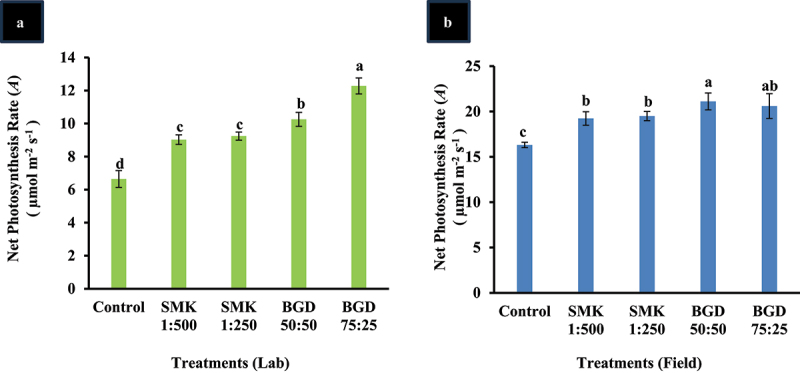
Evaluation of net photosynthesis rate after application of smoke (SMK) and biogas digestate (BGD) slurry. (a) lab conditions and (b) field conditions. The data are expressed as mean ± standard deviation of three replicates. The bars labeled with different alphabets are statistically significantly different at *p < 0.005.*

Plants grown in lab condition were tested for photosynthetic parameters with smoke (SMK) and BGD slurry solutions. Amongst all the parameters related to photosynthesis, the net photosynthesis rate (*A*) was significantly higher in the plants treated with BGD (75:25) i.e. 46% increase followed by BGD (50:50) with 35% as compared to in control, respectively (Figure 1(a)). The plants treated with smoke solution also exhibited significantly higher *A* as compared to control; however, the application of BGD surpassed the smoke solution treatment (Figure 1(a)).

Among all treated plants, an insignificant increase was observed for intercellular CO_2_ (C_i_) (Supp. Figure 1(a)). Transpiration rate (E) was significantly higher in BGD 50:50 as compared to the control (Supp. Figure 1(b)). For other treatments, E was not significantly different from the control. The stomatal conductance (g_s_) was also found to be insignificantly different from control (Supp. Figure 1(c)).

The plants grown in the open field were also treated with BGD and SMK and analyzed for the *A*. Like lab conditions, the level of *A* was statistically higher in all treatments as compared to the control (Figure 1(b)). However, *A* was higher in BGD-treated plants as compared to the smoke solution. The highest level was found in BGD 50:50 (23% increase) and BGD 75:25 (21% increase) than the control (Figure 1(b)).

The intercellular CO_2_ (C_i_) was attenuated in SMK 1:250 treated plants in comparison with control and other treatments (Supp. Figure S1(d)). In BGD 75:25, the highest level of C_i_ was observed. No significant variation was observed for transpiration and stomatal conductance (Supp. Figure S1(e,f).

### Water use efficiency (WUE) enhanced in SMK- and BGD-treated plants

**Figure 2.**
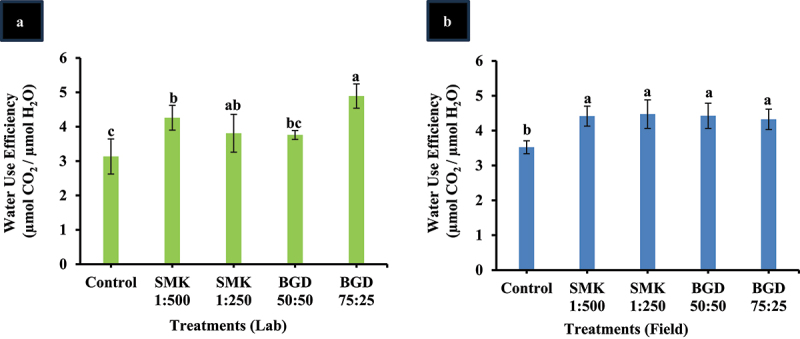
Water use efficiency after SMK and BGD treatment. (a) lab conditions and (b) field conditions. The data are expressed as mean ± standard deviation of three replicates. The bars labeled with different alphabets are statistically significantly different at *p < 0.005.*

Next the water use efficiency (WUE) was measured for lab-treated plants, and a significant increase was observed for higher concentration of BGD 75:25 and SMK 1:250. For BGD 75:25, the increase of 36% and in SMK 1:250 26% enhancement was observed than the control (Figure 2(a)). In the other two treatments, i.e. BGD (50:50) and SMK (1:500), a non-significant increase was observed than control (Figure 2(a)).

In field-grown plants, WUE was also significantly higher in all treatments as compared to control (Figure 2(b)). Smoke solution SMK 1:250 depicted the highest WUE (21%) followed by BGD 50:50 (20%) than the control (Figure 2(b)). It is important to mention that WUE in all the treatments was statistically similar.

### Enhancement in total carotenoids and chlorophyll content of treated plants

**Figure 3.**
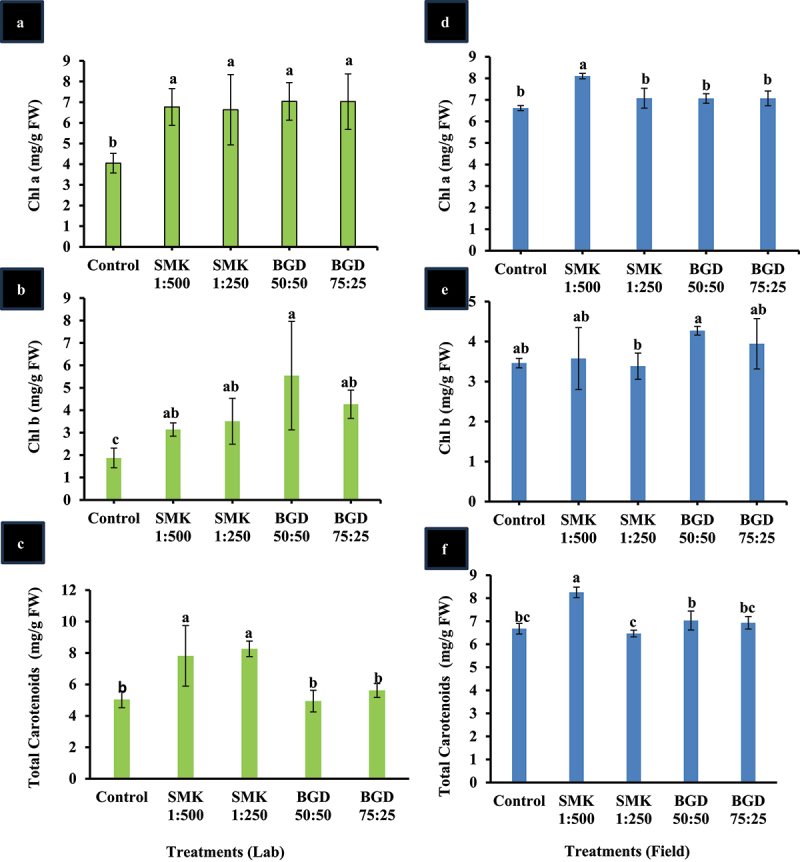
Analysis of pigments in SMK- and BGD-treated plants. (a) Chl a in lab conditions, (b) Chl b in lab conditions, (c) carotenoids in lab conditions, (d) Chl a in field conditions, (e) Chl b in field conditions, (f) carotenoids in field conditions. The data are expressed as mean ± standard deviation of three replicates. The bars labeled with different alphabets are statistically significantly different at *p < 0.005.*

For lab-treated plants, next chlorophyll a (Chl a), chlorophyll b (Chl b), and total carotenoids were measured (Figure 3). All the treated plants exhibited significantly higher Chl a level as compared to control plants. Among the treated plants, BGD application exhibited higher levels though they were not statistically significant than smoke application. BGD-treated plants exhibited a 42% increase in Chl a while the smoke-treated plants depicted nearly 40% increase (Figure 3(a)). The BGD-treated plants showed significantly higher Chl b content as compared to control and SMK 1:250 treated plants (Figure 3(b)). For BGD 50:50 42% while BGD 75:25 25% increase was found in chl b levels (Figure 3(b)). Contrary to chlorophyll content, the total carotenoids level was significantly higher in SMK-treated plants; however, the BGD-treated plants exhibited higher levels than control but statistically insignificant (Figure 3(c)). In SMK 1:250 and SMK 1:500, 40% and 37% increase were observed, respectively.

In field-treated plants, the pattern of Chl a, Chl b, and total carotenoids levels was not consistent with the lab-treated plants (Figure 3(d–f)). Chl a was significantly higher in SMK 1:500 than control and other treated plants and depicted nearly 18% increase as compared to control (Figure 3(d)). Although other treated plants depicted higher levels of Chl a than control, these levels were statistically insignificant. Chl b level was not significantly higher in all treated plants as compared to control (Figure 3(e)). Total carotenoids content was highest in SMK 1:500 as compared to control and other treated plants (Figure 3(f)). In SMK 1:500, a 20% higher level was observed as compared to control (Figure 3(f)).

### Improvement in growth parameters of treated plants

**Figure 4.**
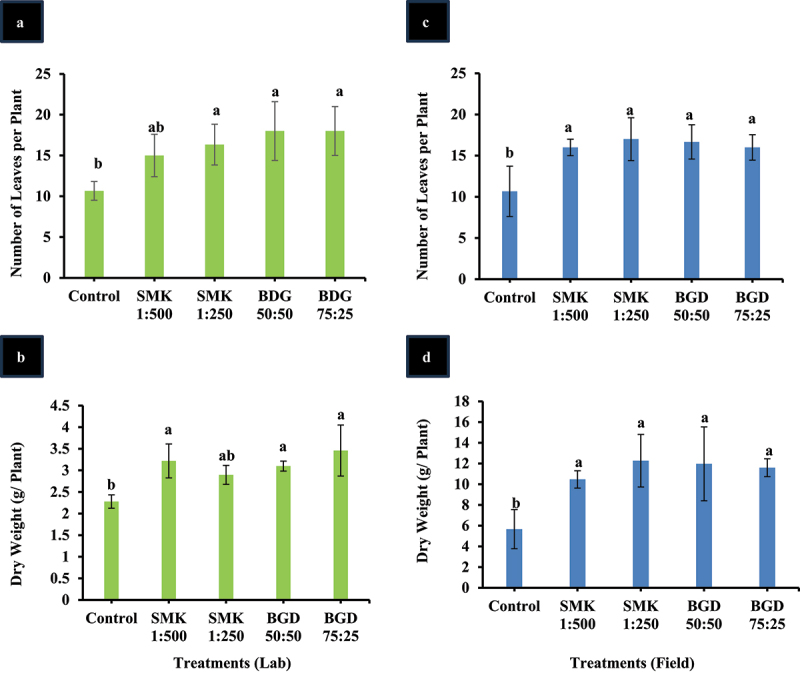
Analysis of growth parameters in SMK- and BGD-treated plants. (a) Leaf number in lab conditions, (b) dry weight in lab conditions, (c) leaf number in field conditions, (d) dry weight in field conditions. The data are expressed as mean ± standard deviation of three replicates. The bars labeled with different alphabets are statistically significantly different at *p < 0.005.*

Next, the growth parameters including leaf number and dry weight were measured in treated plants under lab conditions (Figure 4). BGD and SMK 1:250 treated plants leaf number was statistically higher than the control (Figure 4(a)). SMK 1:500 treated plants showed statistically comparable results as compared to other treated plants. It is important to mention that SMK 1:500 plants exhibited an insignificant increase of 28% higher leaf number than control (Figure 4(a)). Plant dry weight was higher in all treated plants as compared to control. Among the treated plants, the SMK 1:250 treatment showed higher but insignificant increase as compared to control (Figure 4(b)). BGD 75:25 treatment showed the highest dry weight as compared to all other plants, and the level was 34% higher than control (Figure 4(b)).

In field-treated plants, as compared to control, all treated plants exhibited significantly higher leaf number (Figure 4(c)). Among all treatments, the leaf number were statistically insignificantly different; however, highest number (17) was found in SMK1:250. With reference to control, SMK 1:250 depicted 37% higher leaf number (Figure 4(c)). Dry weight was also found to be higher in all treated plants as compared to control (Figure 4(d)). Consistent to leaf number, SMK 1:250 depicted higher plant dry weight (48%) as compared to control (Figure 4(d)).

For lab grown plants, leaf area was significantly higher in BGD 75:25 in comparison with control but this increase was not significantly different from other treatments (Supp. Figure S2(a)). In the field-grown plants, an insignificant increase was observed in leaf area as compared to the control (Supp. Figure S2(b)). Tuber weight was significantly higher in all treatments as compared to the control. However, the highest weight was observed in SMK 1:500 (495 g per plant) followed by BGD 75:25 (473 g per plant) (Supp. Figure S2(c)).

### Augmentation of total sugars in the treated plants

**Figure 5.**
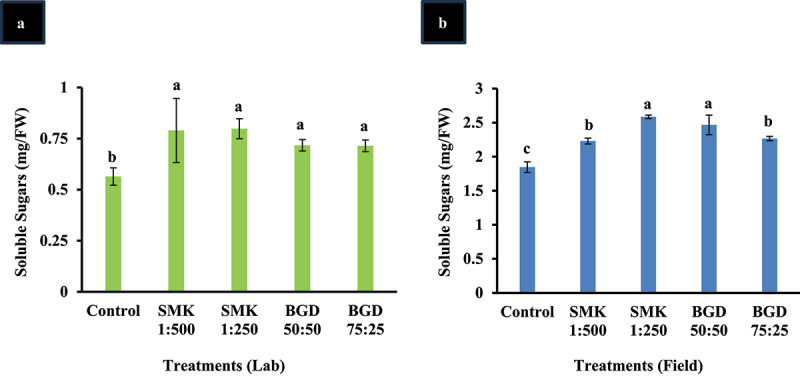
Enhancement in soluble sugars after SMK and BGD treatment. (a) lab conditions and (b) field conditions. The data are expressed as mean ± standard deviation of three replicates. The bars labeled with different alphabets are statistically significantly different at *p < 0.005.*

In lab-treated plants, soluble sugars were found to be statistically higher than control (Figure 5(a)). The SMK-treated plants showed the highest level of soluble sugars. The highest level of nearly 30% increase was found in SMK-treated plants as compared to control (Figure 5(a)).

For field-treated plants, total soluble sugars were found significantly higher in all the treatment groups (Figure 5(b)). However, highest concentration was observed in SMK 1:250 (28%) followed by BGD 50:50 (25%) as compared to other treatments and control plants (Figure 5(b)).

### Principal component analysis (PCA) of treated plants

For traits measured under lab and field conditions, PCA analysis was performed wherein all the treatments showed similar trend as compared to control group (Figure 6(a,c)). However, in lab conditions, scatteredness was found in treatments while in field one of the replicates, SMK 1:250 was separately grouped as outlier in the PCA analysis (Figure 6(a,c)).

**Figure 6.**
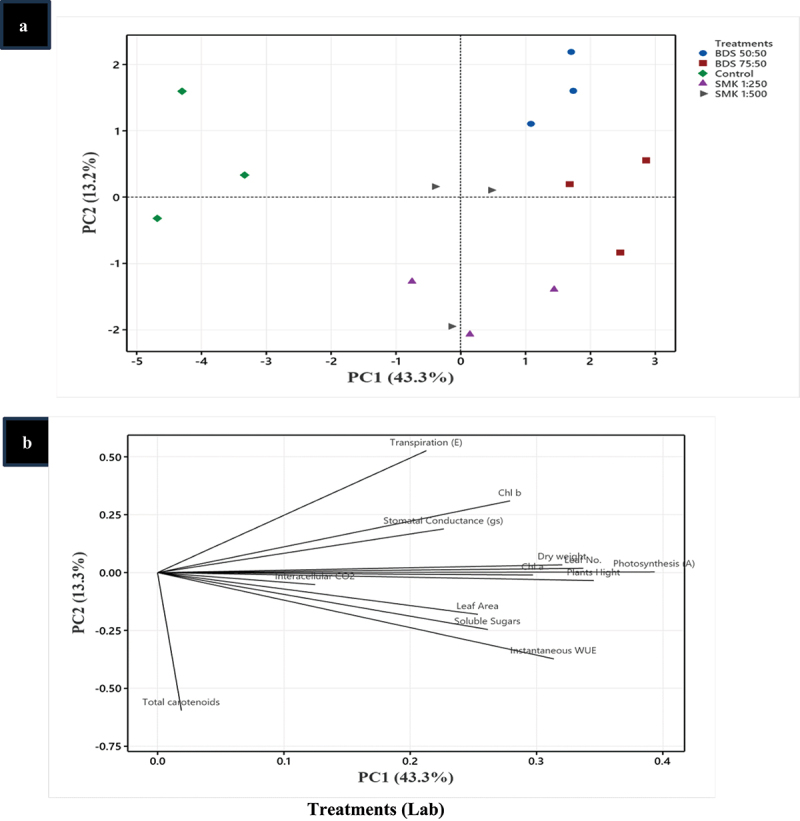
Principal component analysis (PCA) of SMK- and BGD-treated plants. (a) Treatments in lab conditions, (b) treatment responses in lab conditions, (c) treatments in in field conditions, (d) treatment responses in field conditions.

The PCA analysis showed that after the treatments (lab conditions), except total carotenoids, parameters like photosynthesis, Chl a, dry weight, and leaf number indicated the highest contribution toward plant growth (Figure 6(b)). In field conditions, a similar trend was observed wherein photosynthesis and soluble sugars also contributed in improving plant growth (Figure 6(d)).

**Figure 6.**
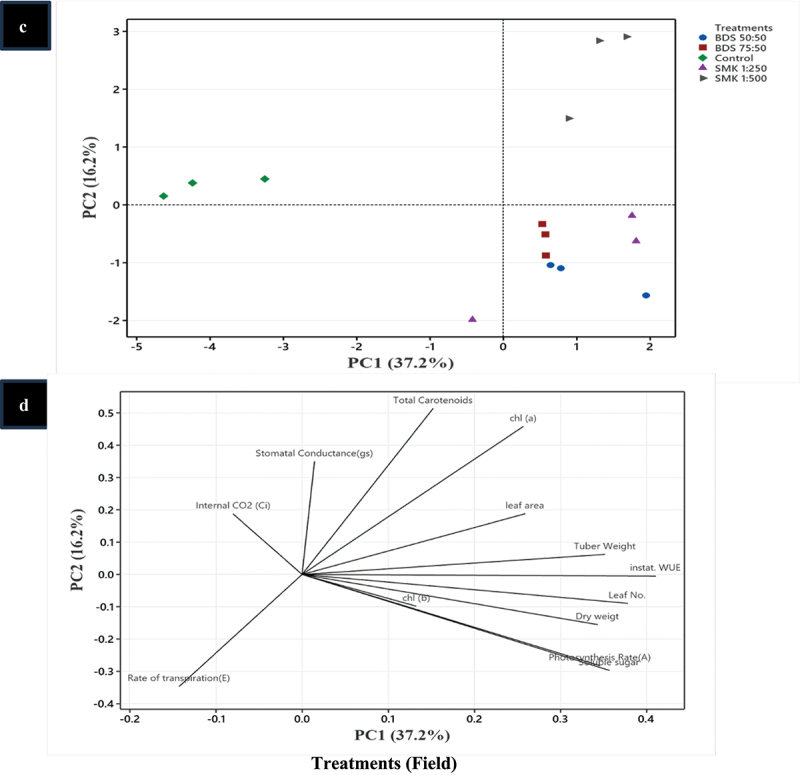
(Continued).

### DNA methylation analysis of genomic DNA after exposure to different treatments of smoke and slurry

To study the effect of different treatments of smoke and slurry on the DNA methylation profile of potato plants at all the 5’-CCGG-3’ across the whole genome, three primer combinations were used. The scoring of amplified bands revealed 40 bands. Under control conditions, 20 non-methylated bands (represented by bands present in both *Eco*RI/*Hpa*II and *Eco*RI/*Msp*I sample and denoted as type I) were observed, 18 methylated bands which were absent in both *Eco*RI/*Msp*I and *Eco*RI/*Hpa*II sample (denoted as type II), no fully methylated bands were observed indicated by the presence of band only in the *Eco*RI/*Msp*I sample (type III), and two hemi-methylated bands were observed indicated by the presence of band only in the *Eco*RI/*Hpa*II sample (type IV). In smoke treatments, 23 and 11 non-methylated bands (type I), 17 and 22 methylated bands (Type II), 0 and 1 fully methylated bands (Type III), and 0 and 6 hemi-methylated bands (type IV) were observed in SMK 1:500 and SMK 1:250, respectively (Table 2). In BGD slurry treatments, 7 and 4 non-methylated bands (type I), 14 and 29 methylated bands (Type II), 9 and 1 fully methylated bands (Type III), and 10 and 6 hemi-methylated bands (type IV) were observed, respectively (Table 2).

**Table 2.** Methylation profiling of control and treatments.

Plant Name						T Met Ratio %	Full Met Ratio %	Hemi Met Ratio %	Un-Met Ratio %
	Type	Type	Type	Type					
	I	II	III	IV	SUM				
C	20	18	0	2	40	50	45	5	50
SMK1	23	17	0	0	40	42.5	42.5	0	57.5
SMK2	11	22	1	6	40	72.5	57.5	15	27.5
BGD1	7	14	9	10	40	82.5	57.5	25	17.5
BGD2	4	29	1	6	40	90	75	15	10

As indicated from the data (Table 2) that the smoke and slurry treatments are influencing the DNA methylation profile differently showing the irrelevance in the molecular mechanism between the two treatments, the DNA methylation of these treatments will be explained separately. Comparison of different smoke treatments to control showed that bands showing total methylated ratio decreased from 50% in control to 42.5% in SMK1:500 and increased from 50% in control to 72.5% in SMK 1:250. In comparison with the control, the two concentrations of smoke treatments affected the DNA methylation profile very differently indicating the significant effect of the concentration of smoke on plant regulation. Comparison of different concentrations of slurry treatments to control showed that total methylated ratio increased from 50% in control to 82.5% in BGD 50:50 and 90% in BGD 75:25. This shows that BGD treatment caused an increase in DNA methylation ratio.

### Dynamics of methylation/demethylation events in relation to different concentrations of smoke and slurry

In order to identify the changes in the methylation and demethylation events, comparative MSAP profiling was done by scoring all banding patterns between the control and different concentrations of smoke and slurry (Table 3). This comparison revealed that SMK 1:500 showed high level of conservation in DNA methylation pattern as compared to the control as 92.5% events showed no change in DNA methylation pattern between control and SMK 1:500 and 7.5% demethylation events and no methylation events as compared to control. The pattern showed a drastic shift in SMK 1:250 where 62.5% showed no change in DNA methylation pattern between control and SMK 1:250, 10% demethylation events and 27.5% methylation events as compared to control. The comparison between control and slurry revealed that BGD 50:50 showed 35% conservation in DNA methylation pattern as compared to the control, 30% demethylation events and 35% DNA methylation events as compared to control. BGD 75:25 showed 55% of events with no change in DNA methylation pattern compared to control, no DNA demethylation events, and 45% DNA methylation events as compared to control.

**Table 3.** Comparison of methylation dynamics of control vs treatments.

Response	C vs SMK1	C vs SMK2	C vs BGD1	C vs BGD2
No Change	92.5	62.5	35	55
DeMethylation	7.5	10	30	0
Methylation	0	27.5	35	45

### Identification of differential bands

**Figure 7.**
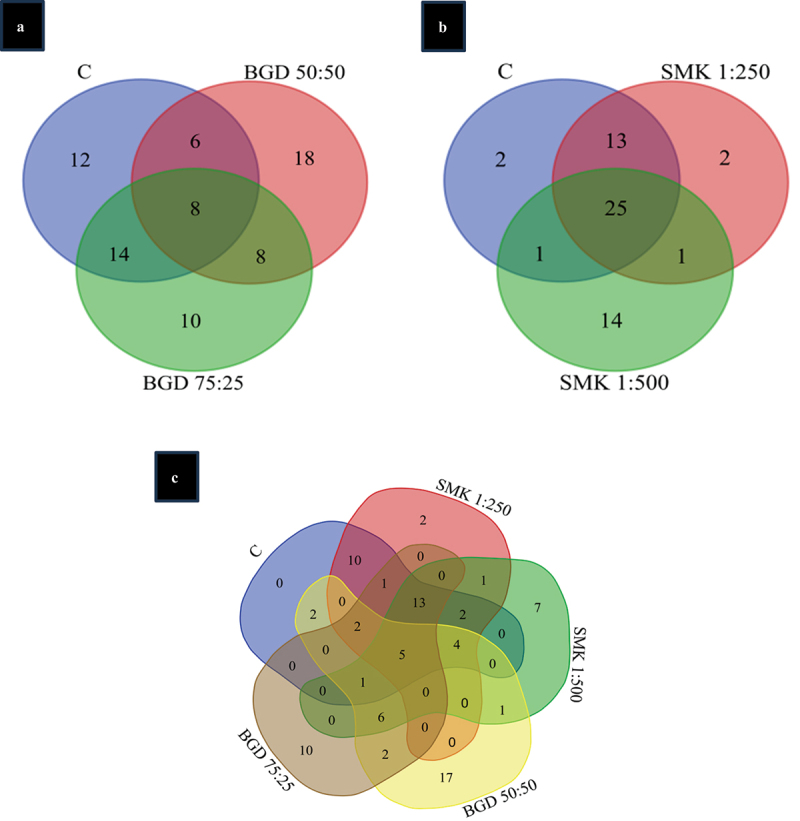
Venn diagram of SMK and BGD treatments. (a) Venn diagram of BGD treatment, (b) venn diagram of smoke treatment, figure 7C: venn diagram of all treatments.

To identify the common and unique band among control and different concentrations of smoke and slurry, Venn diagram analysis was performed. In smoke treatments, 25 common bands were shared by control, SMK 1:500 and SMK 1:250 treatments, 13 bands were common between control and SMK 1:500, whereas only 2 unique bands were present in SMK 1:500. Interestingly, only 1 band was found to be common between control and SMK 1:250, while 14 unique bands were found in SMK 1:250 (Figure 7(b)).

In slurry treatments, 8 common bands were shared between control, BGD 50:50 and BGD 75:25 treatments, 6 bands were common between control and BGD 50:50, whereas 18 unique bands were present in BGD 50:50. BGD 75:25 have 14 bands common between control and BGD 75:25, while 10 unique bands were found in BGD 75:25 (Figure 7(a)).

The Venn diagram analysis of all the treatments and control revealed 2 unique bands in SMK 1:500, 7 in SMK 1:250, 17 in BGD 50:50, and 10 in BGD 75:25 indicating that the BGD treatment is causing more unique bands as compared to SMK treatment (Figure 7(c)).

## Discussion

Improvement in agriculture under changing climate is the prime target to feed the rising population.^1^ One of the challenges in improving crop productivity is the ever increasing prices of synthetic fertilizers and their hazardous effect on environment.^2^ This warrants the need to use biostimulants or biofertilizers that can boost the plant productivity.^4^ Different biostimulants, such as amino acids and hydrolyzates, were obtained from plants, animals, microorganisms, and industrial by-products that when applied improved the plant growth.^1,9^ In this study, exogenous application of plant-derived smoke (SMK) and BGD slurry on potato is reported that improved the photosynthetic parameters, pigments, growth, and altered genome-wide methylation profile.

Initially, it was established that plant-derived smoke solutions promote seed germination and seedling growth.^34^ Smoke-derived solutions contain karrikins (KAR) that act as positive growth stimulator.^35^ In various studies, the efficacy of smoke in stimulating germination and seedling vigor has been reported in different plants such as bean, maize, papaya, wheat, tomato, and okra.^36,37^ It was further reported that the plant-derived smoke can also alter other parameters such as photosynthesis, flowering, nutritional value, and yield in plants.^38–40^ In our study, plants grown in lab and field when evaluated after the application of smoke depicted increase in net photosynthesis rate (*A*) (Figure 1). This result is in line with the reported studies about efficiency of smoke solution on plant photosynthesis.^35,40^

However, contrary to photosynthesis, the other parameters like intercellular CO_2_ (C_i_), transpiration rate (E), and stomatal conductance (g_s_) were not significantly different from control. These results are contradictory to earlier reports of Akeel *et al.*, 2019, which might be due to different source of plant-derived smoke and the plant species under investigation. However, in our study, the water use efficiency (WUE) was improved. This can be attributed to the unaffected E and g_s_. In previous studies, improvement in g_s_ and E has been reported along with increased stomatal opening, though this increase is positive, but it can lead to excessive water loss, thus, reducing WUE.

Plant pigments such as Chl a, Chl b, and carotenoids play vital role in photosynthesis.^41^ The Chl a and carotenoids were higher in plants after smoke treatment (Figure 3). However, the Chl b content was not improved (Figure 3). In field conditions, SMK 1:500 treatment resulted in higher Chl a and carotenoid contents (Figure 3). It has been reported that less concentrated smoke solution is more effective in elevating these pigments.^15,19^ In mint plant, improvement in chlorophyll content due to foliar application of smoke solution has been reported.^42^ In another study, elevation in chlorophyll content of wheat has been reported after treatment with smoke solution.^43^ The smoke solution has reportedly improved physiological and biochemical parameters of wheat.^44^ Chlorophyll and carotenoids are involved in light harvesting in photosynthesis and improvement in their content have been reported to improve net photosynthesis.^41^ Carotenoids are also important component of anti-oxidative system in plants and increased carotenoids are reported to improve different abiotic stresses.^45^ Therefore, smoke acts not only as growth stimulator but also as stress protecting agent.

Enhancement in dry weight was observed in smoke-treated plants (Figure 4,) which can be attributed to the improved net photosynthesis and leaf number. The increase in leaf number can be linked to the improved plant height (lab grown plants, data not shown). However, despite the increase in leaf number, leaf area remains unaffected. Furthermore, soluble sugars were improved in SMK-treated plants that also can be attributed to the increased net photosynthesis. Improvement in height of tomato plant after the application of smoke has been reported.^39^ Other studies also reported improvement in plant growth parameters and dry weight.^17,19,46^ In field-grown plants, tuber yield was considerably improved in smoke-treated plants. Higher tuber yield can be attributed to improved photosynthesis and growth parameters. Moreover, higher sugar levels are considered to improve source to sink partitioning of photosynthate in potato, thus increasing the tuber yield.^47,48^

In addition to smoke, BGD slurry can also be used as biostimulant to improve crop productivity. BGD is the by-product of biogas which is produced through anaerobic digestion of crop residues and animal manure.^11,49^ It is a valuable by-product and offers inexpensive source of organic matter, minerals, and nutrients^12^ It contains macronutrients like potassium, nitrogen, and phosphorous as well as micronutrients.^12^ BGD is not a replacement of soil applied synthetic fertilizers; however, the BGD application by foliar means can help provide nutrients directly via aerial plants parts to target and address nutrient deficiency promptly.^50^ It is reported that foliar application improves nutrient use efficiency and reduce soil contamination.^51^ A comprehensive review highlights the efficacy of foliar application in enhancing plant growth and productivity.^50^

In our study, the application of BGD improved photosynthesis rate in both lab- and field-grown plants as compared to the control (Figure 1). WUE was also improved in field-grown plants (Figure 2). Moreover, Chl b was enhanced in field, but the Chl a content was not higher than control (Figure 3). However, in the lab experiment, Chl a and Chl b contents were increased as compared to control (Figure 3). The variation in pigment content in field can be attributed to the fact that under natural conditions more evaporation takes place which might have resulted in less absorption of slurry. Similar trend has been observed in net photosynthesis rate wherein lab-grown plants exhibited higher rate as compared to field-grown plants. Furthermore, the growth-related parameters including leaf number, plant dry weight, and soluble sugars were also enhanced in lab as well as field-grown plants (Figures 4 and 5). It is also important to mention that the tuber weight was also increased after the BGD treatment in field conditions. The increase in these parameters can be linked to the increase in photosynthesis rate. It is considerably established now that the BGD can effectively improve growth parameters in different crop plants such as rice, wheat, barley, maize, peanut, brassica, and ryegrass.^49,52–57^ Alongside macronutrients and micronutrients, BGD also contains humic acid and amino acids.^49^ The nitrogen positively regulates root and shoot growth along with meristematic growth.^58^ Nitrogen is also a major constituent of proteins and enzymes; so it is also involved in modulating the plant metabolism.^58^ Other macronutrients in BGD also help enhance fruit size, plant vigor, and root growth.^13^

Smoke contains KAR resembling the butanolide.^59^ Butanloids regulate plant responses such as cuticle formation, root hair density, and anthocyanin accumulation.^59^ It is well documented that application of smoke solution has altered the gene expression and enzymatic activities in treated plants.^60^ Although smoke application has altered gene expression, the underlying mechanism is not clearly known. For that we performed genome-wide methylation profiling of treated plants.

Total methylation ratio was reduced in SMK 1:500 as compared to control while in SMK 1:250 it was higher than the control. SMK 1:500 depicted 7.5% demethylation, whereas in SMK 1:250 10% demethylation was observed. The DNA methylation/demethylation influences the gene expression profiling.^32^ Alongside DNA methylation is also utilized in defense by controlling the activity of transposable elements.^61^ This finding gives a new insight into the role of smoke solution to regulate plant metabolism. However, in depth analysis can enhance the understanding about the role of smoke solution in growth enhancement and stress protection.

Total methylation trend in BGD 50:50 and BGD 75:25 was concentration-dependent. However, there was difference in demethylation patterns wherein in BGD 50:50 treatment 30% demethylation event was observed. While in BGD 75:25, no DNA methylation was observed. It is reported that DNA methylation is affected by the nitrogen.^62,63^ Epigenetic changes are involved in nitrogen uptake that also regulate energy and photosynthesis-related genes.^63^ In *Arabidopsis*, set domain group 8 is induced by nitrogen application which triggers gene expression reprogramming.^64,65^ Other macronutrients, potassium and phosphorus, are also implicated in alteration of DNA methylation.^66,67^ The application of BGD has supplied the nutrients to the plants which in turn also modulated DNA methylation pattern.

## Conclusion

The results obtained in this study indicate the efficacy of plant-derived smoke and BGD slurry in improving plant performance. Although varied response has been observed in SMK and BGD treatments, both exert control over plant. Moreover, the methylation pattern also varied between two treatments that suggest the independent role of SMK and BGD in regulating plant responses. However, in-depth analysis of gene expression is required to understand the mechanism behind varied methylation profiling. Moreover, combined application of SMK and BGD is required to elucidate a possible synergistic effect on plant performance.

## Supplementary Material

Supplemental Material

## References

[1] Rouphael Y, Colla G. Biostimulants in agriculture. Front Plant Sci. 2020;11:40. Frontiers Media SA. doi:10.3389/fpls.2020.00040. 32117379 PMC7010726

[2] Zerssa GW, Hailemariam M, Tadele KT. Improving the sustainability of agriculture: challenges and opportunities. In: Lousada S, editor. Land-Use Management-Recent Advances, New Perspectives, and Applications. Intech Open; 2023. doi:10.5772/intechopen.112857.

[3] Devi PI, Manjula M, Bhavani RV. Agrochemicals, environment, and human health. Annu Rev Environ Resour. 2022;47(1):399–13. doi:10.1146/annurev-environ-120920-111015.

[4] Kurniawati A, Toth G, Ylivainio K, Toth Z. Opportunities and challenges of bio-based fertilizers utilization for improving soil health. Org Agric. 2023;13(3):335–350. doi:10.1007/s13165-023-00432-7.

[5] Yakhin OI, Lubyanov AA, Yakhin IA, Brown PH. Biostimulants in plant science: a global perspective. Front Plant Sci. 2017;7:2049. doi:10.3389/fpls.2016.02049. 28184225 PMC5266735

[6] Van Oosten MJ, Pepe O, De Pascale S, Silletti S, Maggio A. The role of biostimulants and bioeffectors as alleviators of abiotic stress in crop plants. Chem Biol Technol Agric. 2017;4(1):1–12. doi:10.1186/s40538-017-0089-5.

[7] Xu L, Geelen D. Developing biostimulants from agro-food and industrial by-products. Front Plant Sci. 2018;9:1567. doi:10.3389/fpls.2018.01567. 30425724 PMC6218572

[8] Drobek M, Frąc M, Cybulska J. Plant biostimulants: importance of the quality and yield of horticultural crops and the improvement of plant tolerance to abiotic stress—A review. Agronomy. 2019;9(6):335. doi:10.3390/agronomy9060335.

[9] Behera B, Supraja KV, Paramasivan B. Integrated microalgal biorefinery for the production and application of biostimulants in circular bioeconomy. Bioresour Technol. 2021;339:125588. doi:10.1016/j.biortech.2021.125588. 34298244

[10] Puglia D, Pezzolla D, Gigliotti G, Torre L, Bartucca ML, Del Buono D. The opportunity of valorizing agricultural waste, through its conversion into biostimulants, biofertilizers, and biopolymers. Sustainability. 2021;13(5):2710. doi:10.3390/su13052710.

[11] Funes-Pinter I, Pisi G, Aroca M, Uliarte EM. Compost tea and bioslurry as plant biostimulants. Part 2: biofertilizer test in ornamental flowers. J Plant Nutr. 2023;46(13):3041–3052. doi:10.1080/01904167.2023.2171883.

[12] Yadav R, Sudhishri S, Khanna M, Lal K, Dass A, Kushwaha HL, Bandyopadhyay K, Dey A, Kushwah A, Nag RH. et al. Temporal characterization of biogas slurry: a pre-requisite to sustainable nutrigation in crop production. Front Sustain Food Syst. 2023;7:1234472. doi:10.3389/fsufs.2023.1234472.

[13] You L, Yu S, Liu H, Wang C, Zhou Z, Zhang L, Hu D. Effects of biogas slurry fertilization on fruit economic traits and soil nutrients of camellia oleifera Abel. PloS One. 2019;14(5):e0208289. doi:10.1371/journal.pone.0208289. 31071086 PMC6508714

[14] Zhang X, Wu D, Zakharchenko E, Qn L, A G, F L, Y W, M F. Review on effects of biogas slurry application on crop growth. Explore (New York, NY). 2022;19(4):571–577. doi:10.1016/j.explore.2022.11.001.

[15] Khatoon A, Rehman SU, Aslam MM, Jamil M, Komatsu S. Plant-derived smoke affects biochemical mechanism on plant growth and seed germination. Int J Mol Sci. 2020;21(20):7760. doi:10.3390/ijms21207760. 33092218 PMC7588921

[16] Kulkarni MG, Rengasamy KRR, Pendota SC, Gruz J, Plačková L, Novák O, Doležal K, Van Staden J. Bioactive molecules derived from smoke and seaweed ecklonia maxima showing phytohormone-like activity in Spinacia oleracea L. N Biotechnol. 2019;48:83–89. doi:10.1016/j.nbt.2018.08.004. 30098416

[17] Jamil M, Kanwal, M, Aslam, MM Khan, SU, Malook, I, Tu, J, ur Rehman, S. Effect of plant-derived smoke priming on physiological and biochemical characteristics of rice under salt stress condition. Aust J Crop Sci. 2014;8(2):159–170.

[18] Singh S, Uddin M, Khan MMA, Chishti AS, Singh S, Bhat UH. The role of plant-derived smoke and karrikinolide in abiotic stress mitigation: an omic approach. Plant Stress. 2023;7:100147. doi:10.1016/j.stress.2023.100147.

[19] Shah G, Tu J, Fayyaz M, Masood S, Ullah H, Jamil M. Moringa oleifera smoke induced positive changes in biochemical, metabolic, and antioxidant profile of rice seedling under cadmium stress. Int J Phytorem. 2023;25(10):1337–1347. doi:10.1080/15226514.2022.2157793. 36573355

[20] Pinit S, Ariyakulkiat L, Chaiwanon J. Rice straw-derived smoke water promotes rice root growth under phosphorus deficiency by modulating oxidative stress and photosynthetic gene expression. Sci Rep. 2023;13(1):14802. doi:10.1038/s41598-023-41987-5. 37684292 PMC10491667

[21] Hayat N, Afroz N, Rehman S, Bukhari SH, Iqbal K, Khatoon A, Taimur N, Sakhi S, Ahmad N, Ullah R. et al. Plant-derived smoke ameliorates salt stress in wheat by enhancing expressions of stress-responsive genes and antioxidant enzymatic activity. Agronomy. 2021;12(1):28. doi:10.3390/agronomy12010028.

[22] Murashige T, Skoog F. A revised medium for rapid growth and bio assays with tobacco tissue cultures. Physiol Plant. 1962;15(3):473–497. doi:10.1111/j.1399-3054.1962.tb08052.x.

[23] Kamran M, Imran Q, Khatoon A, Lee I, Rehman S. Effect of plant extracted smoke and reversion of abscisic acid stress on lettuce. Pak J Bot. 2013;45:1541–1549.

[24] Association APH. Standard methods for the examination of water and wastewater. Washington DC, USA: American Public Health Association; 1926.

[25] Odnell A, Recktenwald M, Stensén K, Jonsson B-H, Karlsson M. Activity, life time and effect of hydrolytic enzymes for enhanced biogas production from sludge anaerobic digestion. Water Res. 2016;103:462–471. doi:10.1016/j.watres.2016.07.064. 27498254

[26] Yun S, Xing T, Han F, Shi J, Wang Z, Fan Q, Xu H. Enhanced direct interspecies electron transfer with transition metal oxide accelerants in anaerobic digestion. Bioresour Technol. 2021;320:124294. doi:10.1016/j.biortech.2020.124294. 33129089

[27] Zeb I, Yousaf S, Ali M, Yasmeen A, Khan AZ, Tariq JA, Zhao Q, Abbasi AM, Ahmad R, Khalil TM. et al. In-situ microaeration of anaerobic digester treating buffalo manure for enhanced biogas yield. Renewable Energy. 2022;181:843–850. doi:10.1016/j.renene.2021.09.089.

[28] Afsar S, Bibi G, Ahmad R, Bilal M, Naqvi TA, Baig A, Shah MM, Huang B, Hussain J. Evaluation of salt tolerance in eruca sativa accessions based on morpho-physiological traits. PeerJ. 2020;8:e9749. doi:10.7717/peerj.9749.

[29] Abbasi AZ, Bilal M, Khurshid G, Yiotis C, Zeb I, Hussain J, Baig A, Shah MM, Chaudhary SU, Osborne B. et al. Expression of cyanobacterial genes enhanced CO2 assimilation and biomass production in transgenic Arabidopsis thaliana. PeerJ. 2021;9:e11860. doi:10.7717/peerj.11860. 34434649 PMC8359801

[30] Barickman TC, Kopsell DA, Sams CE. Abscisic acid impacts tomato carotenoids, soluble sugars, and organic acids. HortScience. 2016;51(4):370–376. doi:10.21273/HORTSCI.51.4.370.

[31] Chow PS, Landhäusser SM. A method for routine measurements of total sugar and starch content in woody plant tissues. Tree Physiol. 2004;24(10):1129–1136. doi:10.1093/treephys/24.10.1129. 15294759

[32] Saad M, Mary H, Amjid U, Shabir G, Aslam K, Shah SM, Khan AR. Photoperiodic stress induces genotype-specific shift in DNA methylation in tartary buckwheat. Biol Futur. 2019;70(4):278–285. doi:10.1556/019.70.2019.31. 34554545

[33] Ahmad RC, Bilal M, Jeon J-H, Kim HS, Park Y-I, Shah MM, Kwon S-Y. Improvement of biomass accumulation of potato plants by transformation of cyanobacterial photorespiratory glycolate catabolism pathway genes. Plant Biotechnol Rep. 2016;10(5):269–276. doi:10.1007/s11816-016-0403-x.

[34] Pierce SM, Esler K, Cowling RM. Smoke-induced germination of succulents (mesembryanthemaceae) from fire-prone and fire-free habitats in South Africa. Oecologia. 1995;102(4):520–522. doi:10.1007/BF00341366. 28306897

[35] Akeel A, Khan MMA, Jaleel H, Uddin M. Smoke-saturated water and karrikinolide modulate germination, growth, photosynthesis and nutritional values of carrot (Daucus carota L.). J Plant Growth Regul. 2019;38(4):1387–1401. doi:10.1007/s00344-019-09941-w.

[36] Iqbal M, Asif S, Ilyas N, Raja NI, Hussain M, Shabir S, Ashraf Faz MN, Rauf A. Effect of plant derived smoke on germination and post germination expression of wheat (*triticum aestivum* L.). AJPS. 2016;7(6):806–813. doi:10.4236/ajps.2016.76075.

[37] Kulkarni MG, Ascough GD, Van Staden J. Effects of foliar applications of smoke-water and a smoke-isolated butenolide on seedling growth of okra and tomato. HortScience. 2007;42(1):179–182. doi:10.21273/HORTSCI.42.1.179.

[38] Keeley JE. Smoke-induced flowering in the fire-lily Cyrtanthus ventricosus. South African J Bot. 1993;59(6):638. doi:10.1016/S0254-6299(16)30681-0.

[39] Kulkarni MG, Ascough GD, Van Staden J. Smoke-water and a smoke-isolated butenolide improve growth and yield of tomatoes under greenhouse conditions. Horttechnology. 2008;18(3):449–454. doi:10.21273/HORTTECH.18.3.449.

[40] Zhou J, Fang L, Wang X, Guo L, Huang L. Effects of smoke-water on photosynthetic characteristics of isatis indigotica seedlings. Sustain Agric Res. 2013;2(2):24. doi:10.5539/sar.v2n2p24.

[41] Aremu AO, Kulkarni MG, Bairu MW, Finnie JF, Van Staden J. Growth stimulation effects of smoke-water and vermicompost leachate on greenhouse grown-tissue-cultured ‘Williams’ bananas. Plant Growth Regul. 2012;66(2):111–118. doi:10.1007/s10725-011-9634-6.

[42] Singh M, Uddin M, Chishti AS, Bhat UH, Khan S, Khan MMA. Plant-derived smoke water and karrikinolide (KAR1) enhance physiological activities, essential oil yield and bioactive constituents of mentha arvensis L. Front Plant Sci. 2023;14:1129130. doi:10.3389/fpls.2023.1129130. 37152142 PMC10159057

[43] Shabir S, Ilyas N, Asif S, Iqbal M, Kanwal S, Ali Z. Deciphering the role of plant-derived smoke solution in ameliorating saline stress and improving physiological, biochemical, and growth responses of wheat. J Plant Growth Regul. 2022;41(7):2769–2786. doi:10.1007/s00344-021-10473-5.

[44] Iqbal M, Asif S, Ilyas N, Raja NI, Hussain M, Ejaz M, Saira H. Smoke produced from plants waste material elicits growth of wheat (triticum aestivum L.) by improving morphological, physiological and biochemical activity. *Biotechnol Rep*. 2018;17:35–44. doi:10.1016/j.btre.2017.12.001. PMC5735255 29270367

[45] Swapnil P, Meena M, Singh SK, Dhuldhaj UP, Marwal A, Marwal A. Vital roles of carotenoids in plants and humans to deteriorate stress with its structure, biosynthesis, metabolic engineering and functional aspects. Curr Plant Biol. 2021;26:100203. doi:10.1016/j.cpb.2021.100203.

[46] Waheed MA, Jamil M, Khan MD, Shakir SK, Rehman SU. Effect of plant-derived smoke solutions on physiological and biochemical attributes of maize (zea mays L.) under salt stress. Pak J Bot. 2016;48:1763–1774.

[47] Viola R, Roberts AG, Haupt S, Gazzani S, Hancock RD, Marmiroli N, Machray GC, Oparka KJ. Tuberization in potato involves a switch from apoplastic to symplastic phloem unloading. Plant Cell. 2001;13(2):385–398. doi:10.1105/tpc.13.2.385. 11226192 PMC102249

[48] Gong H-L, Dusengemungu L, Igiraneza C, Rukundo P. Molecular regulation of potato tuber dormancy and sprouting: a mini-review. Plant Biotechnol Rep. 2021;15(4):417–434. doi:10.1007/s11816-021-00689-y.

[49] Xu W, Zhu Y, Wang X, Ji L, Wang H, Yao L, Lin C. The effect of biogas slurry application on biomass production and forage quality of lolium multiflorum. Sustainability. 2021;13(7):3605. doi:10.3390/su13073605.

[50] Niu J, Liu C, Huang M, Liu K, Yan D. Effects of foliar fertilization: a review of current status and future perspectives. J Soil Sci Plant Nutr. 2021;21(1):104–118. doi:10.1007/s42729-020-00346-3.

[51] Otálora G, Piñero MC, López-Marín J, Varó P, Del Amor FM. Effects of foliar nitrogen fertilization on the phenolic, mineral, and amino acid composition of escarole (cichorium endivia L. var. latifolium). Sci Hortic. 2018;239:87–92. doi:10.1016/j.scienta.2018.05.031.

[52] Hou H, Zhou S, Hosomi M, Toyota K, Yosimura K, Mutou Y, Nisimura T, Takayanagi M, Motobayashi T. Ammonia emissions from anaerobically-digested slurry and chemical fertilizer applied to flooded forage rice. Water Air Soil Pollut. 2007;183(1–4):37–48. doi:10.1007/s11270-007-9353-9.

[53] Garg RN, Pathak H, Das DK, Tomar RK. Use of flyash and biogas slurry for improving wheat yield and physical properties of soil. Environ Monit Assess. 2005;107(1–3):1–9. doi:10.1007/s10661-005-2021-x. 16418901

[54] Terhoeven-Urselmans T, Scheller E, Raubuch M, Ludwig B, Joergensen RG. CO2 evolution and N mineralization after biogas slurry application in the field and its yield effects on spring barley. Appl Soil Ecol. 2009;42(3):297–302. doi:10.1016/j.apsoil.2009.05.012.

[55] Svoboda N, Taube F, Wienforth B, Kluß C, Kage H, Herrmann A. Nitrogen leaching losses after biogas residue application to maize. Soil Tillage Res. 2013;130:69–80. doi:10.1016/j.still.2013.02.006.

[56] Zheng X, Fan J, Cui J, Wang Y, Zhou J, Ye M, Sun M. Effects of biogas slurry application on peanut yield, soil nutrients, carbon storage, and microbial activity in an ultisol soil in southern China. J Soils Sediments. 2016;16(2):449–460. doi:10.1007/s11368-015-1254-8.

[57] Zhu K, Choi HL, Yao HQ, Suresh A, Oh DI. Effects of anaerobically digested pig slurry application on runoff and leachate. Chem Ecol. 2009;25(5):359–369. doi:10.1080/02757540903193114.

[58] Islam MR, Rahman SME, Rahman MM, Oh DH, Ra CS. The effects of biogas slurry on the production and quality of maize fodder. Turkish J Agric For. 2010;34(1):91–99. doi:10.3906/tar-0902-44.

[59] Tian H, Watanabe Y, Nguyen KH, Tran CD, Abdelrahman M, Liang X, Xu K, Sepulveda C, Mostofa MG, Van Ha C. et al. KARRIKIN UPREGULATED F-BOX 1 negatively regulates drought tolerance in arabidopsis. Plant Physiol. 2022;190(4):2671–2687. doi:10.1093/plphys/kiac336. 35822606 PMC9706471

[60] Aslam MM, Khatoon A, Jamil M, Ur Rehman S, Komatsu S. Plant-derived smoke solution: a stress alleviator in crop. J Plant Growth Regul. 2024;1–18. doi:10.1007/s00344-023-11221-7.

[61] Zhou W, Liang G, Molloy PL, Jones PA. DNA methylation enables transposable element-driven genome expansion. Proc Natl Acad Sci. 2020;117(32):19359–19366. doi:10.1073/pnas.1921719117. 32719115 PMC7431005

[62] Mager S, Ludewig U. Massive loss of DNA methylation in nitrogen-, but not in phosphorus-deficient zea mays roots is poorly correlated with gene expression differences. Front Plant Sci. 2018;9:497. doi:10.3389/fpls.2018.00497. 29725341 PMC5917015

[63] Zhang H, Zhang X, Xiao J. Epigenetic regulation of nitrogen signaling and adaptation in plants. Plants. 2023;12(14):2725. doi:10.3390/plants12142725. 37514337 PMC10386408

[64] Gifford ML, Dean A, Gutierrez RA, Coruzzi GM, Birnbaum KD. Cell-specific nitrogen responses mediate developmental plasticity. Proc Natl Acad Sci. 2008;105(2):803–808. doi:10.1073/pnas.0709559105. 18180456 PMC2206617

[65] Li Y, Brooks M, Yeoh-Wang J, McCoy RM, Rock TM, Pasquino A, Moon CI, Patrick RM, Tanurdzic M, Ruffel S. et al. SDG8-mediated histone methylation and RNA processing function in the response to nitrate signaling. Plant Physiol. 2020;182(1):215–227. doi:10.1104/pp.19.00682. 31641075 PMC6945839

[66] Tian P, Lin Z, Lin D, Dong S, Huang J, Huang T. The pattern of DNA methylation alteration, and its association with the changes of gene expression and alternative splicing during phosphate starvation in tomato. Plant Journal. 2021;108(3):841–858. doi:10.1111/tpj.15486. 34492142

[67] Labra M, Grassi F, Imazio S, Di Fabio T, Citterio S, Sgorbati S, Agradi E. Genetic and DNA-methylation changes induced by potassium dichromate in Brassica napus L. Chemosphere. 2004;54(8):1049–1058. doi:10.1016/j.chemosphere.2003.10.024. 14664833

