# Supplementary material for: Stimulatory effects of smoke solution and biogas digestate slurry application on photosynthesis, growth, and methylation profiling of solanum tuberosum
Source: Plant Signal Behav. 2024 Apr 10;19(1):2336724. doi: 10.1080/15592324.2024.2336724 (PMC11017950; doi:10.1080/15592324.2024.2336724)
Supplement: Supplemental Material [file KPSB_A_2336724_SM8685.pptx]

## Slide 1
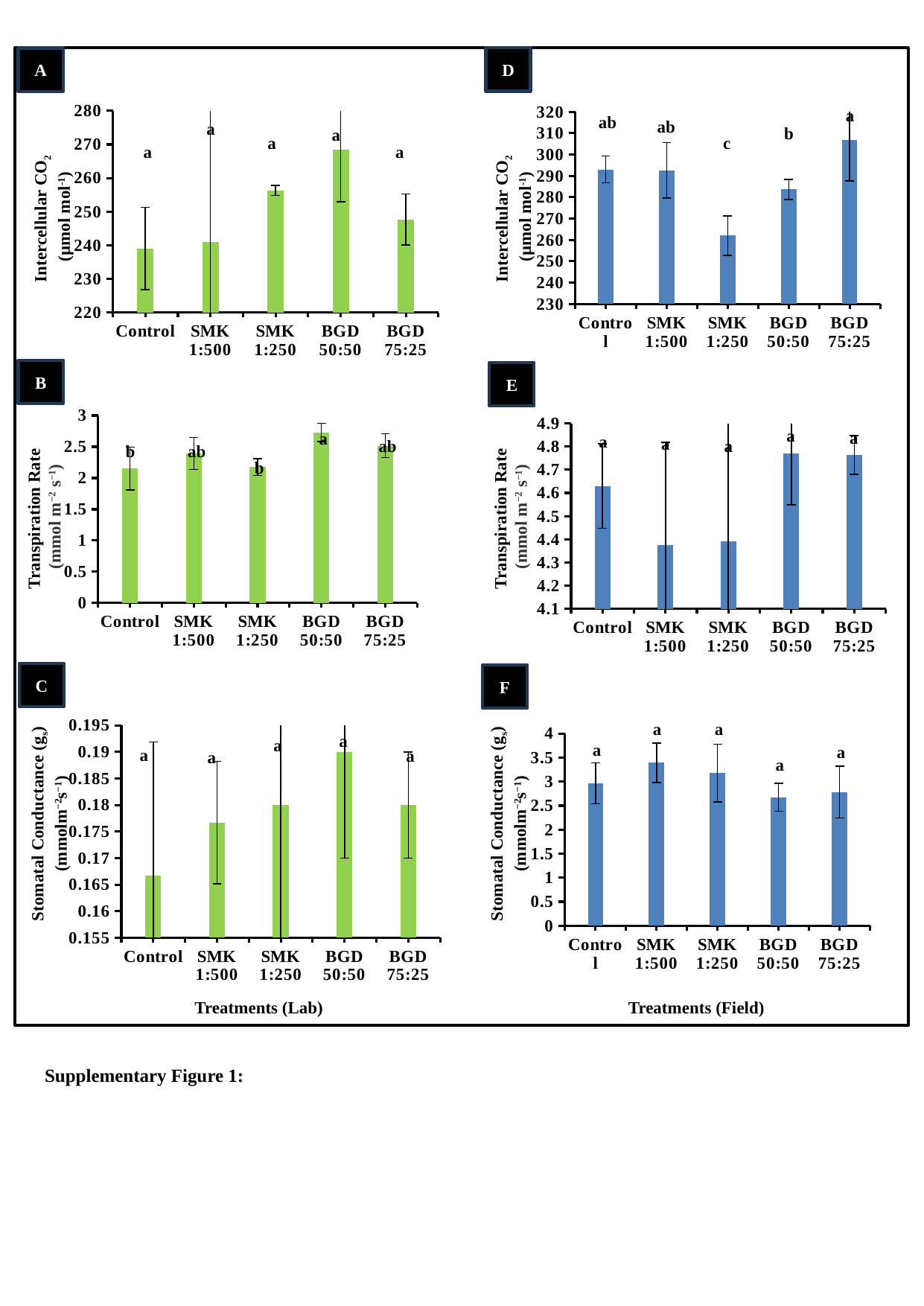

D
A
Intercellular CO2
 (µmol mol-1)
Intercellular CO2
 (µmol mol-1)
### Chart
| Category | |
|---|---|
| Control | 239.0 |
| SMK 1:500 | 241.0 |
| SMK 1:250 | 256.33333329999994 |
| BGD 50:50 | 268.33333329999994 |
| BGD 75:25 | 247.66666669999998 |
### Chart
| Category | |
|---|---|
| Control | 293.0 |
| SMK 1:500 | 292.6666667 |
| SMK 1:250 | 262.0 |
| BGD 50:50 | 283.6666667 |
| BGD 75:25 | 307.0 |a
 ab
ab
a
b
 a
a
c
 a
 a
B
E
Transpiration Rate
(mmol m−2 s−1)
Transpiration Rate
(mmol m−2 s−1)
### Chart
| Category | |
|---|---|
| Control | 2.153333333 |
| SMK 1:500 | 2.3933333329999997 |
| SMK 1:250 | 2.1766666669999997 |
| BGD 50:50 | 2.73 |
| BGD 75:25 | 2.5166666669999995 |
### Chart
| Category | |
|---|---|
| Control | 4.63 |
| SMK 1:500 | 4.373333333 |
| SMK 1:250 | 4.39 |
| BGD 50:50 | 4.77 |
| BGD 75:25 | 4.763333333 |a
a
 a
 a
a
 ab
a
b
 ab
 b
C
F
Stomatal Conductance (gs) (mmolm−2s−1)
Stomatal Conductance (gs) (mmolm−2s−1)
### Chart
| Category | |
|---|---|
| Control | 0.166666667 |
| SMK 1:500 | 0.176666667 |
| SMK 1:250 | 0.18000000000000002 |
| BGD 50:50 | 0.19000000000000003 |
| BGD 75:25 | 0.18000000000000002 |
### Chart
| Category | |
|---|---|
| Control | 2.963333333 |
| SMK 1:500 | 3.3899999999999997 |
| SMK 1:250 | 3.18 |
| BGD 50:50 | 2.673333333 |
| BGD 75:25 | 2.7833333330000007 | a
 a
 a
 a
 a
 a
 a
 a
 a
 a
Treatments (Lab)
Treatments (Field)
Supplementary Figure 1:

## Slide 2
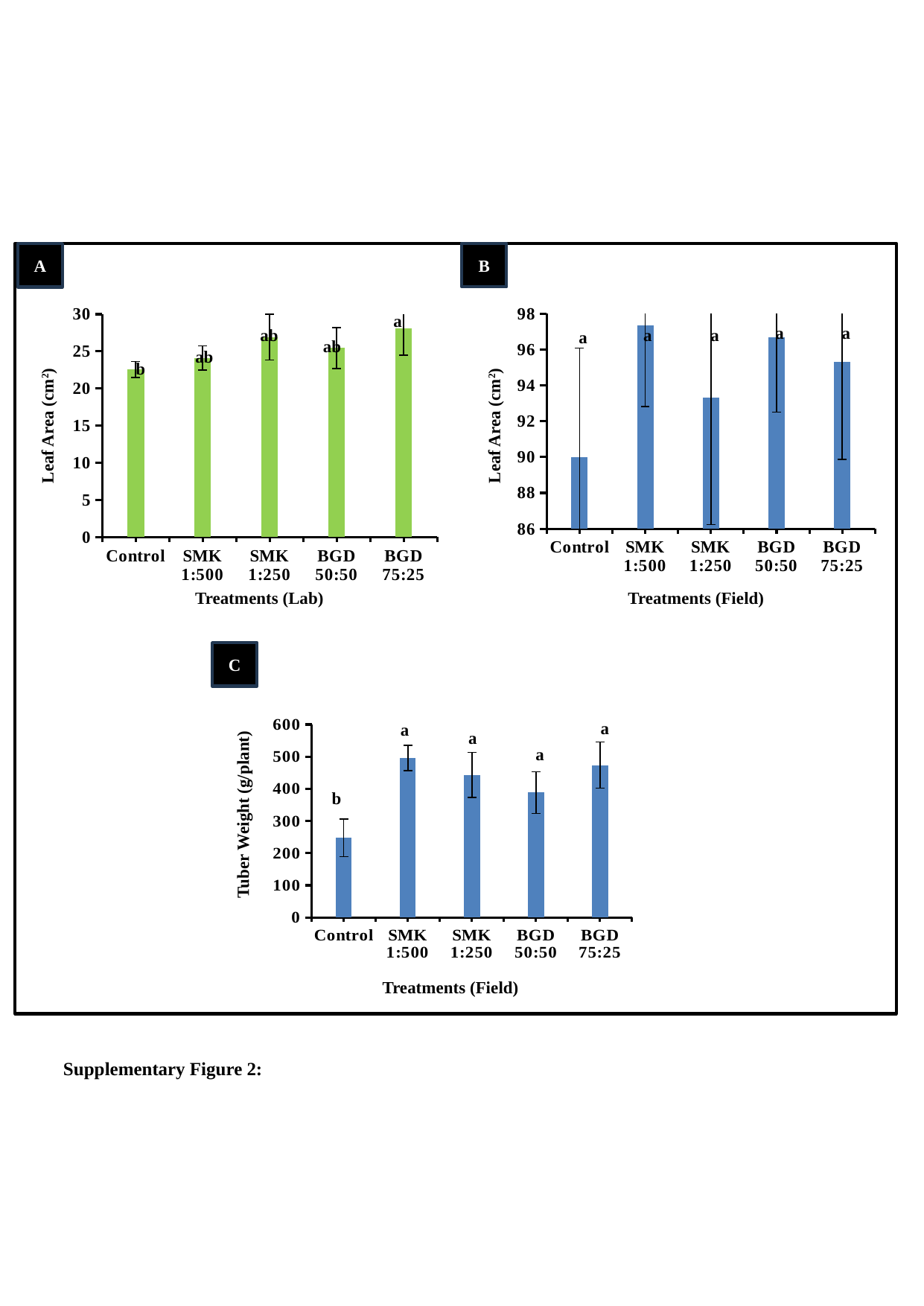

B
A
Leaf Area (cm2)
Leaf Area (cm2)
### Chart
| Category | |
|---|---|
| Control | 22.56667 |
| SMK 1:500 | 24.1 |
| SMK 1:250 | 26.9 |
| BGD 50:50 | 25.43333 |
| BGD 75:25 | 28.13333 |
### Chart
| Category | |
|---|---|
| Control | 90.0 |
| SMK 1:500 | 97.33333333 |
| SMK 1:250 | 93.33333333 |
| BGD 50:50 | 96.66666667 |
| BGD 75:25 | 95.33333333 | a
 a
 a
a
 a
 ab
 a
 ab
 ab
 b
Treatments (Lab)
Treatments (Field)
C
Tuber Weight (g/plant)
### Chart
| Category | |
|---|---|
| Control | 247.93 |
| SMK 1:500 | 495.88 |
| SMK 1:250 | 443.18 |
| BGD 50:50 | 388.1366667 |
| BGD 75:25 | 473.59666669999996 | a
 a
 a
 a
 b
Treatments (Field)
Supplementary Figure 2:
